# Parenting a child with disability: A mother’s reflection on the significance of social support

**DOI:** 10.4102/ajod.v12i0.1157

**Published:** 2023-05-19

**Authors:** Marubini C. Sadiki

**Affiliations:** 1 Department of Inclusive Education, College of Education, University of South Africa, Pretoria, South Africa

## Introduction

This paper is a reflection on my experiences. Here, I reflect on the significance of social support groups as a mother of a child with disabilities. I share my experiences of how I was empowered by the social support of mothers of children with disabilities, with the intention that these experiences will assist other mothers in a rural context. I relate my personal positive experiences of how collaborating with other mothers in the support groups enabled me to be a better parent to my child. This paper presents the significance of coming together as mothers of children with disabilities in a rural setting. I reflect on the implications of raising a child with a disability without social support, and how I overcame those challenges after joining the social support group for mothers of children with disabilities.

## Contextualising this paper

Social support has been identified as an important factor that contributes positively to the caregivers’ (parent, mother) well-being (Lei & Kantor 2021). Tigere and Makhubele (2019) assert that parents of children with disabilities in Sekhukhune District found that support groups reduced anxiety. In Tanzania, McNally and Mannan (2013) observed that the support groups for mothers of children with disabilities were established to help them share their feelings in an open and supportive environment. Dodge et al. (2022) reveal that social support is one of the coping mechanisms used by mothers to cope with raising a child with Down syndrome.

The support groups for mothers of children with disabilities are aimed at aiding mothers during acute periods of stress and change such as: following a birth, initial diagnosis or in response to a major medical intervention (Cauda-Laufer 2017). The inspiring statements of support groups encourage parents to contribute their experiences and views (Cabiati 2021). The support group is a platform to share, guide and learn from other mothers’ experiences of acceptance, enabling mothers of children with disabilities manage their feelings of isolation and loneliness (Cauda-Laufer 2017). The shared experiences of other parents helped mothers think positively about the destiny of their child (Cauda-Laufer 2017). The social interaction and social structures in these groups provide emotional support to mothers of children with disabilities (Dehghan et al. 2022).

Parenting a child with disabilities adversely impacts ones well-being in various ways. These parents may experience: (1) societal stereotypes, (2) prejudices, (3) stigma and (4) psychological health struggles, (5) economic difficulties, and (6) a lack of social and family support. Research conducted in Tanzania reported that caregivers’ experiences of a lack of support contributed to isolation (McNally & Mannan 2013). Parenting a child with a disability may be painful and difficult journey without support – something it could be devastating and challenging to some parents (Cauda-Laufer 2017).

## The challenges I experienced before I was introduced to a support group

Raising a child with disabilities came with significant impact. It overwhelmed me with stress, shock, anger, grief, frustration, embarrassment, worry, disappointment and depression. I felt ashamed of my child. I used to ask myself constantly: ‘Why did I give birth to this kind of a child?; What was the cause?; Is it God’s punishment?; Am I bewitched?’. It was not easy at all; I had no life. I could not go anywhere. I did not have enough time to be social because I had no one to help me. The experiences of parenting a child with a disability are painful. Having to navigate the lonely space is excruciating (Sadiki et al. 2022).

There was no psychological support and I avoided public areas because I did not want the community to know that my child was disabled and I was shy. I experienced hardships without any form of emotional and social support. Especially in moments when families and close colleagues were unsupportive. This insufficient psychological support created distress while parenting a child with disability. Many parents experience a great deal of emotional stress and burnout when taking care of their children (Tigere & Makhubele 2019). I had no social or emotional support when raising my child and I avoided public places to keep him away from the community.

## A sense of belonging in the social support group: My life changed forever

In 1993, I was introduced to a support group of mothers of children with disabilities by a woman who was a member of the Disabled People South Africa (DPSA). Meeting with other parents of children with disabilities helped me experience less stress and gain confidence through sharing parenting experiences. My encounter with the support groups restored my self-esteem, dignity and confidence. This echoes with Tigere and Makhubele (2019), who maintained that support groups are instrumental in giving psychosocial support to parents of children living with disabilities. The support helped me to normalise the experiences of parenting my child with disabilities. This improved my self-image and the upbringing of my child. The support group had a significant advantage as it provided me with opportunities to express my experiences in a mutually supportive manner. It was a platform to share, guide and learn from other mothers’ experiences. I was equipped with knowledge and skills to advocate for my son’s rights and seek services for him.

Sharing my experience with other mothers played a major role in my self-confidence. For example, I became aware of the care dependency grant for children with disabilities through sharing my hardships with other mothers. This openness was therapeutic because we were sharing common matters that affected our wellbeing and the rights of our children. Working together in support groups was an opportunity to share difficulties experienced when mothering a child with disabilities. It was also an intervention to overcome challenges. My participation in the support groups enabled me to engage in self-advocacy and self-representation. During networking, I realised that I was not the only mother, with a child with disabilities, who had challenges, and that I can be of some assistance to others too. This led to my voluntary participation within rural community development initiatives, such as capacity building workshops, creating disability advocacy forums and offering mentoring activities to mothers of children with disabilities.

I am pleased to be an active agent mother of a child with a disability and dedicated to advocate for the rights of children with disabilities. I am a disability inclusion activist. I salute the existence of support groups for the role they played in my empowerment through the parent’s empowerment workshops, training, seminars and conferences. I was among the first group in 2003 to register for a postgraduate programme in disability studies to further empower myself and deepen the advocacy in the group. This exposure enabled me to benefit from participation in international disability and human rights development forums in countries like Canada, the Netherlands, Australia, Lesotho, Norway, and Namibia. These sessions have undoubtedly contributed to the richness of my knowledge on disability rights matters. The exposure also created a platform to further the promotion of disability inclusion. Strengthening partnerships with other disability organisations and parent structures can play a significant role in addressing societal attitudes.

## Conclusion

The positive attitudes from support groups was one of the mechanisms that enabled me to cope with my daily tasks. I know more about my child and I have acquired a wealth of knowledge from the support groups. I have learned that participating in support groups enabled me to have a clear understanding of disability legislations frameworks. I can advocate and lobby for the rights of children with disabilities without fear. According to Pedro, Goldschmidt and Daniels (2019), support groups are instrumental for equipping mothers with helpful information to gain confidence. I encourage mothers to collectively advocate for the rights of children with disabilities through support groups.

The Department of Social Development has an important role in the formation of support groups for parents of children with disabilities (Tigere & Makhubele 2019). Disability advocacy organisations must champion disability awareness rights of parents and children with disabilities in communities. The perceived social support and resilience can significantly reduce the stress of parenting a child with disability in most contexts. It is desired that this article will stimulate future research to contribute to the significance of support groups, and my experiences of parenting a child with disability. My experiences will inspire other mothers to participate in support groups and affiliate to organisations of parents of children with disabilities or lead such groups.
